# A LASSO-Based Method for Detecting Item-Trait Patterns of Replenished Items in Multidimensional Computerized Adaptive Testing

**DOI:** 10.3389/fpsyg.2019.01944

**Published:** 2019-08-30

**Authors:** Jianan Sun, Ziwen Ye

**Affiliations:** Department of Mathematics, College of Science, Beijing Forestry University, Beijing, China

**Keywords:** multidimensional computerized adaptive testing, multidimensional two parameter logistic model, replenished items, item-trait pattern recognition, variable selection, least absolute shrinkage and selection operator, Bayesian information criterion

## Abstract

Multidimensional computerized adaptive testing (MCAT) is one of the widely discussed topics in psychometrics. Within the context of item replenishment in MCAT, it is important to identify the item-trait pattern for each replenished item, which indicates the set of the latent traits that are measured by each replenished item in the item pool. We propose a pattern recognition method based on the least absolute shrinkage and selection operator (LASSO) to detect the optimal item-trait patterns of the replenished items via an MCAT test. Simulation studies are conducted to investigate the performance of the proposed method in pattern recognition accuracy under different conditions across various latent trait correlation, item discrimination, test lengths, and item selection criteria in the test. Results show that the proposed method can accurately and efficiently identify the item-trait patterns of the replenished items in both the two-dimensional and three-dimensional item pools.

## Introduction

Computerized adaptive testing (e.g., Wainer, 2000; Wainer and Mislevy, 2000) based on multidimensional item response theory (MIRT; e.g., Embretson and Reise, 2000; Reckase, 2009) has received much attention in psychometrics during the last few decades. For the pretest of the replenished items in MCAT, recent research mainly focuses on developing online calibration methods (e.g., Chen and Wang, 2016; Chen, 2017; Chen et al., 2017). However, little research has been devoted to discovering the appropriate set of the latent traits that are closely associated with each replenished item, especially from the perspective of assessing the goodness-of-fit for the item with alternative patterns in the MIRT model. For simplicity, the term of item-trait pattern recognition is used to refer to that problem in this research. As a matter of fact, the misspecification of item-trait patterns can produce the risk of lack of fit for MIRT models, which may consequently lead to erroneous individual assessment (e.g., Reckase, 2009; Sun et al., 2016). Besides the existing online calibration methods (e.g., Chen and Wang, 2016; Chen et al., 2017) that have made a remarkable contribution to the calibration of the replenished items in MCAT, identifying item-trait patterns of replenished items is beneficial to the topic of item calibration or pretest by improving the interpretability of the replenished items.

The latent traits of psychological tests are often defined as latent variables in MIRT. The identification of the item-trait patterns of replenished items in MCAT can be formulated as the variable selection problem, which is usually discussed in the field of statistical pattern recognition (e.g., Bishop, 2006; Hastie et al., 2009). The least absolute shrinkage and selection operator (LASSO; Tibshirani, 1996) is deemed one of the most popular variable selection methods. The LASSO is originally constructed on linear regression models and generalized linear models. It is later applied to other models like the Cox proportional hazards models and advances the development in survival analysis, life science and so on (Bishop, 2006; Hastie et al., 2015). For the multidimensional paper-pencil test, Sun et al. (2016) bring forward a latent variable selection method for the MIRT models via the LASSO, which is a recent exploration for item-trait pattern recognition in the paper-pencil test based on MIRT. In that research, they assume the latent traits are entirely unknown. Distinct from the paper-pencil test, the advantage of the MCAT test in conveniently collecting the response and ability information of examinees should be sufficiently utilized for solving the pattern recognition problem.

Note that although the two types of methods, the existing online calibration methods (e.g., Chen and Wang, 2016; Chen et al., 2017) and the proposed pattern recognition method in this research, can both utilize the online feature of the MCAT test, they focus on the item calibration problem from quite different aspects: for each replenished item in the MCAT item pool, the former emphasizes the estimation accuracy of the item parameters for a fitted MIRT model according to a well-known or default pattern, while the latter takes interest in selecting an optimal pattern from all the possible ones for the MIRT model.

The rest of the paper is organized as follows. The compensatory multidimensional item response theory model and the method for variable selection in regression analysis, the LASSO, are briefly introduced in section Compensatory MIRT Model and the LASSO. The original LASSO-based Pattern Recognition Method (LPRM), proposed for detecting the optimal patterns of the replenished items based on the LASSO in MCAT, is described in detail in the third section. In the fourth and fifth sections, the design and results of the simulation studies are presented to evaluate the performance of the proposed pattern recognition method. Conclusion and discussion are summarized in the last section.

## Compensatory MIRT Model and the LASSO

### Compensatory Multidimensional Item Response Theory Model

Assume that there are *N* examinees taking a multidimensional test with *J* items measuring *K* latent traits. The probability of the *i*th examinee with the ability vector **θ**_*i*_ correctly answering the *j*th item is defined by the compensatory multidimensional two-parameter model (Reckase, 2009):

(1) $$\begin{array}{l} P\left(Y_{ij}=1|\theta_{i},a_{j},b_{j}\right)=F\left({a_{j}}^{T}\theta_{i}+b_{j}\right), \end{array}$$

where *Y*_*ij*_ = 1 indicates the correct response of the *i*th examinee to the *j*th item; *Y*_*ij*_ = 0 indicates the incorrect response. The ability parameters of the *i*th examinee are denoted as the *K*-dimensional vector **θ**_*i*_ = (θ_*i*__1_, …, θ_*iK*)_^T^. The discrimination parameters of the *j*th item are denoted as the *K*-dimensional vector ***a***_*j*_ = (*a*_*j*__1_, …, *a*_*jK*)_^T^. The intercept parameter of the *j*th item is denoted as *b*_*j*_. If the cumulative distribution function *F* in Equation (1) is specified as the standard logistic function, the multidimensional two-parameter logistic model (M2PLM; Reckase, 2009) is obtained:

(2) $$\begin{array}{l} P\left(Y_{ij}=1|\theta_{i},a_{j},b_{j}\right)=\frac{\exp\left({a_{j}}^{T}\theta_{i}+b_{j}\right)}{1+\exp\left({a_{j}}^{T}\theta_{i}+b_{j}\right)}. \end{array}$$

### LASSO-A Variable Selection Method for Regression Models via L_1_ Regularization

The LASSO is originally proposed in regression analysis in order to select variables for linear models and generalized linear models via L_1_ regularization. Different from traditional search approaches such as the forward selection, backward elimination and stepwise selection in regression analysis, the sparsity of the regression coefficients produced by the LASSO can improve the prediction accuracy and interpretability of the regression models (e.g., Bishop, 2006; Hastie et al., 2009). As pointed by Hastie et al. (2015), the LASSO or L_1_-regularized regression depending on the L_1_ norm of the regression coefficient vector yields a convex optimization problem for variable selection; furthermore, the algorithm of coordinate descent (Fu, 1998) is especially fast for the LASSO with linear models because the coordinate-wise minimizers are explicitly available, and an iterative search along each coordinate is not needed.

The binary-response logistic model is a popular generalization of linear models. Note that if the latent traits are assumed to be known, denoted by the matrix Θ = _(_θ_*ik*_)*N*_ × *K*_, the M2PLM for a given item reduces to the binary-response logistic model. The LASSO or the named L_1_-regularized optimization for that model (Tibshirani, 1996; Friedman et al., 2010) with the *j*th item is formulated as

(3) $$\begin{array}{l} \underset{b_{j},a_{j1},\cdots \;,a_{jK}}{\min}\left\{-l\left(b_{j},a_{j1},\ldots ,a_{jK};Y_{j},\Theta \right)+\lambda \overset{K}{\underset{k=1}{\sum }}|a_{jk}|\right\}, \end{array}$$

where *l*(*b*_*j*_, *a*_*j*__1_, …, *a*_*jK*_) is the log-likelihood function for the observed data ***Y***_*j*_ = (*Y*_1j_, …, *Y*_*Nj*_)^T^ and the known abilities Θ. The L_1_ norm of the discrimination parameters for item *j* (i.e., ***a***_*j*)_ is formulated by $\overset{K}{\underset{k=1}{\sum^{\;}}}|a_{jk}|$. The tuning parameter or regularization parameter is denoted by λ, which takes non-negative values and controls the sparsity of ***a***_*j*_. In Equation (3), as long as the λ value is sufficiently large, the L_1_-penalty mainly dominates the optimization and leads to the sparsity of ***â***_*j*_. For instance, if λ = ∞, ***â***_*j*_ is an all-zeros vector. In addition, λ = 0 delivers the solution to the ordinary least-squares problem; that is, Equation (3) reduces to the regular optimization for estimating the parameters of item *j* without any shrinkage effect. If λ is positive and not large enough, the solution to Equation (3) yields sparse ***a***_*j*_ vectors, which has only some nonzero coordinates.

The objective in Equation (3) is convex and the likelihood part is differentiable, so finding a solution is a standard task in convex optimization. Both the algorithm of pathwise coordinate descent (Friedman et al., 2007, 2010) and the algorithm of cyclical coordinate descent (Hastie et al., 2015) are concise and efficient for the L_1_-regularized optimization (3). Note that the M2PLM based on the well-known Θ reduces to the binary-response logistic model, the optimization formulated by Equation (3), consequently, can be solved by the cyclical coordinate descent directly.

In order to obtain the optimal variable selection result, it is necessary to set an appropriate value for λ; however, it is not intuitive. A common approach is to construct a group of the L_1_-regularized optimizations for a range of λ values and apply the cross-validation (e.g., Devijver and Kittler, 1982; Efron, 1983; Bishop, 2006; Hastie et al., 2009) or information criterion like the Bayesian information criterion (BIC; Schwarz, 1978) to choose the optimal solution from the alternatives. Although cross-validation can analyze the prediction errors of solution paths, its major disadvantage is the high computational complexity (Bishop, 2006), whereas the information criterion like the BIC can eliminate that disadvantage (e.g., Sun et al., 2016).

It should be clarified that the problem of the item-trait pattern recognition for the item replenishment in MCAT is significantly distinct from that of the existing online calibration methods, such as Chen and Wang (2016) and Chen et al. (2017). The former pays close attention to the interpretability and concision of the item-trait patterns for MIRT models, while the latter puts much emphasis on the calibration or estimation of the replenished items, which aims to conveniently get accurate estimates for item parameters via the MCAT procedure, basically assuming that each item is measured by all the *K* latent traits or the patterns are well-known beforehand. Note that in practice, each replenished item is often associated with only a subset of the *K* traits. Although the patterns of the replenished items might be determined by treating the small loadings as zero, it is inevitable to bring about the risk of misspecification of the patterns from the lack of fit for the MIRT models. Thus, the variable selection approaches discussed in the pattern recognition issue could provide a reasonable way to resolve the problem. For instance, for the paper-pencil multidimensional test, Sun et al. (2016) introduce an approach developed on the LASSO to identify the item-trait patterns for several binary compensatory MIRT models, which can select latent variables for the models via the L_1_-regularized regressions and get the optimal pattern via the BIC. The ability parameters of the MIRT models in that research are assumed to be unknown and deemed nuisance parameters, which are therefore eliminated by applying the expectation-maximization (EM) algorithm.

## A LASSO-based Method for Item-trait Pattern Recognition in MCAT

The aim of this research is to detect the optimal item-trait patterns of the replenished items in the MCAT item pool based on the LASSO. Without loss of generality, it is assumed that the number of ability dimensions is much smaller than the examinee sample size, which is a usual scenario in many ordinary educational tests and helps to ensure the performance of the LASSO (e.g., Hastie et al., 2009). In the research of Sun et al. (2016), the ability parameter **θ** is deemed unknown for the paper-pencil test and therefore the missing data treatment is necessary. Nevertheless, if $\hat{\theta }$ substitutes for **θ** in the M2PLM, the model is reduced to a binary-response generalized linear logistic model. It means that the LASSO for the generalized linear model (Friedman et al., 2010) can be extended to solve the pattern recognition problem. The essential responses and ability estimates of examinees can be conveniently obtained in an MCAT test, which could improve the computing efficiency and save the costs of the data collection process for pattern recognition. Also, mixing the replenished items into the operational ones in the MCAT test can automatically put the item parameters of the replenished items on the same scale as the operational ones. Thus, the three-step method based on the LASSO, referred to as the LPRM, is developed for detecting the optimal item-trait patterns of replenished items as follows.

Step 1. Assume that an MCAT item pool measures *K* latent traits and consists of *J*_0_ operational items and *J*_1_ replenished items. Organize *N* examinees to take an MCAT test. It has *Z*_0_ operational items and *Z*_1_ replenished items: the former is chosen among the *J*_0_ operational items in the item pool by a given item selection method in MCAT. The latter is designated among the *J*_1_ replenished items in the item pool. Specifically, since *J*_1_ is relatively large in practice, it can be designed that each of the *J*_1_ replenished items is answered by a sub-sample of examinees: *Z*_1_ takes an appropriate value that is smaller than *J*_1_ so that the test length is acceptable for the corresponding examinees. Although different examinees may answer different replenished items, it is not difficult to ensure that enough responses to each replenished item are recorded via an appropriate design. For instance, if *J*_1_ = 30 and *N* = 2,000, all the examinees can be divided into 5 groups. Among each group, 400 examinees were designated to answer *Z*_1_ = 6 replenished items. The replenished items are also assumed to be consistent with the operational items so that the examinees are not capable of distinguishing them, which makes sure the examinees share the same test motivation for the two types of items. In addition, the essential parts of the MCAT test such as the item selection method and the stopping rule can be designed to follow regular settings. Thus, the response data for the chosen operational and replenished items as well as the estimated ability parameters are recorded in the MCAT test: all the examinees' responses to the operational items are scored and used for getting their ability estimates, $\hat{\Theta }$; all the responses to the replenished items are scored and prepared for the optimizations in the next step.

Step 2. For each given item, set a group of values for λ, as denoted by λ_1_, …, λ_*W*_, and set up the corresponding L_1_-regularized optimizations for different λ values. For instance, the L_1_-regularized optimization for the *j*th replenished item (*j* = 1, …, *J*_1_) based on a fixed λ_*w*_ is constructed by

(4) $$\begin{array}{l} \underset{b_{j},a_{j1},\cdots \;,a_{jK}}{\min}\left\{-l\left(b_{j},a_{j1},\ldots ,a_{jK};Y_{j},\hat{\Theta }\right)+\lambda_{w}\overset{K}{\underset{k=1}{\sum }}|a_{jk}|\right\}, \end{array}$$

where ***Y***_*j*_ is the binary vector of the responses to the *j*th item. The matrix $\hat{\Theta }$ is the ability estimates of the corresponding examinees. The algorithm of cyclical coordinate descent or pathwise coordinate descent can be used here to solve the optimizations for the given λ_1_, …, λ_*W*_. The essential part of the corresponding optimizations is to get the estimated discrimination parameters, ${â}_{j}^{\;\left(1\right)}$, …, ${â}_{j}^{\;\left(W\right)}$ and then the alternative or candidate item-trait patterns for the *j*th item. Here the true pattern of the *j*th item is denoted by the *K*-dimensional vector ***Q***_*j*_ = (*q*_*j*__1_, …, *q*_*jK*)_^T^. If the *k*th latent trait is measured by that item, *q*_*jk*_ = 1; else, *q*_*jk*_ = 0. Thus, the alternative patterns for the *j*th item obtained from the optimizations within λ_*1*_, …, λ_*W*_ are denoted as vectors ${\hat{Q}}_{j1}$, …, ${\hat{Q}}_{jW}$, respectively. Take the solution to Equation (4) at the given λ_*w*_ as an example: the sparsity of the estimates ${â}_{j}^{\;\left(w\right)}$ is treated as the *w*th alternative pattern for the *j*th item, ${\hat{Q}}_{jw}$, which refers to a *K*-dimensional vector with 0 or 1. That is, if ${â}_{j,k_{\text{1}}}^{\left(w\right)}=\ldots ={â}_{j,k_{t}}^{\left(w\right)}=0$, the *k*_1_th, …, *k*_*t*_th traits are not measured by item *j*, and therefore the vector ${\hat{Q}}_{jw}$ has 0 on the *k*_1_th, …, *k*_*t*_th dimensions and has 1 on the rest dimensions. The optimal item-trait pattern of the *j*th item is denoted by the vector ${\hat{Q}}_{j}^{*}$ = ${\left(q_{j1}^{*},\;\ldots ,\;q_{jK}^{*}\right)}^{T}.$

Step 3. Apply the BIC to choose the optimal item-trait patterns for the replenished items from the alternative patterns. Specifically, the goodness-of-fit for the M2PLM to the data can be appropriately measured by the BIC: the well-penalized likelihood avoids the over-fit effect (e.g., Burnham et al., 2002) that is produced by the parameter redundancy of the model (e.g., Sun et al., 2016). The BIC is therefore implemented for finding the optimal patterns of the replenished items from the alternatives. The decision rule is minimizing the BIC according to the alternative pattern combinations for different λ values (i.e., λ_1_, …, λ_*W*_): $\left\{{\hat{Q}}_{j}^{*}\right\}$ prefers the combination of patterns admitting the minimum value among *BIC*_1_, …, *BIC*_*W*_.

Note that Step 3 serves for the purpose of detecting item-trait patterns rather than estimating item parameters directly. The solution in terms of *a* and *b* parameters to the Equation (4) indeed suffers the shrinkage effect, which is produced by the L_1_-penalty of the optimization, so those estimates should not be used as the estimates for *a* and *b* parameters within the context of MIRT parameter calibration. It needs to be clarified that the calibration of *a* and *b* parameters for *J*_1_ replenished items in the item pool should be re-estimated according to the detected item-trait patterns, $\left\{{\hat{Q}}_{j}^{*}\right\}$ (*j* = 1, …, *J*_1_), which are regarded as known for the M2PLM after implementing the proposed method. The treatment here can be deemed a special case of the simplified relaxed LASSO (e.g., Meinshausen, 2007; Hastie et al., 2017). Since the responses to the replenished items have been collected, the further task for the item calibration or item parameter estimation can follow a regular approach like the confirmatory factor analysis for MIRT models (e.g., Reckase, 2009) or the existing online calibration methods (e.g., Chen and Wang, 2016; Chen et al., 2017).

## Simulation for the LPRM With the Fixed-Length MCAT

Studies 1 and 2 were conducted corresponding to the two-dimensional and three-dimensional MCAT item pools, individually, to explore the performance of the LPRM with the fixed-length test in discovering the optimal item-trait patterns of the replenished items. For each of the two studies, different conditions across various latent trait correlation, item discrimination, test lengths, and item selection criteria were designed. For each condition, 50 datasets were generated as the replications in the simulation for enhancing the generality of the results. Computing codes were written in the R software.

### Item Pool and Data Design

#### Item Pool Generation

For each of the two studies, two levels of discrimination parameters (i.e., {*a*_*jk*_}, *j* = 1, …, J_*_; *k* = 1, …, *K*; J_*_ = *J*_0_ + *J*_1_) corresponding to two types of item pools were generated. One type of item pool had moderately discriminating items: the *a* parameters were drawn from the uniform distribution, *U*(0.7, 1.3). The other type of item pool had highly discriminating items: the *a* parameters were drawn from the uniform distribution, *U*(1.1, 1.7). For both the two types of item pool, the intercept parameters (i.e., *b*_1_, …, $b_{J^{*}}$) were drawn from the standard normal distribution.

In Study 1, each of the two-dimensional item pools had *J*_0_ = 900 operational items and 3 types of patterns were produced. Specifically, 300 items measured the first trait; another 300 items measured the second trait; the other 300 items measured both two traits. Each item pool in Study 1 also had *J*_1_ = 30 replenished items. Among those, there were 10 items measured the first trait, another 10 items measured the second trait, and the rest 10 items measured both two traits. It was assumed that three latent traits were measured by each item pool in Study 2 and the 7 types of patterns were involved. Each item pool in Study 2 consisted of *J*_0_ = 910 operational items and *J*_1_ = 35 replenished items, every 130 operational items and every 5 replenished items of which corresponded to one of the seven types of patterns.

#### Response Data Generation

For Studies 1 and 2, the sample size of the examinees was *N* = 2,000. The latent traits were assumed to be independent and correlated, individually. Specifically, the true ability parameters were generated from the multivariate normal distribution with the mean of 0s and two covariance matrices, individually. In Study 1, the covariance matrices were designed as $\left(\begin{array}{cc} 1 & 0\\ 0 & 1 \end{array}\right)$ and $\left(\begin{array}{cc} 1 & 0.3\\ 0.3 & 1 \end{array}\right)$. In Study 2, the covariance matrices were $\left(\begin{array}{ccc} 1 & 0 & 0\\ 0 & 1 & 0\\ 0 & 0 & 1 \end{array}\right)$ and $\left(\begin{array}{rrr} 1 & 0.2 & 0.3\\ 0.2 & 1 & 0.5\\ 0.3 & 0.5 & 1 \end{array}\right)$. The examinees' responses to each item in the item pool were generated from the M2PLM with the true ability parameters and the item parameters.

### The Procedure of Item-Trait Pattern Recognition by the LPRM

The MCAT test required by Step 1 of the LPRM, as mentioned in section A LASSO-based Method for Item-Trait Pattern Recognition in MCAT, was simulated via the package of mirtCAT (Chalmers, 2016) in the R software. The ability estimates of the examinees based on the M2PLM were obtained by the maximum a posteriori (MAP) in the simulated MCAT. Those estimates were regarded as the inputs of the latent traits for the L_1_-regularized optimization in Step 2 of the LPRM. The essential responses of the examinees to the replenished items required by the LPRM were also obtained by the MCAT test. The details of the test in Step 1 of the LPRM are illustrated as follows.

#### Item Selection Methods in MCAT

Both in Studies 1 and 2, the operational items for each examinee were selected from the item pool by two item selection criteria: D-optimality and Bayesian A-optimality (Segall, 1996, 2000; van der Linden, 1999; Mulder and van der Linden, 2009). D-optimality selects the *k*th item by

(5) $$\begin{array}{l} \underset{i_{k}\in R_{k}}{\arg\max}\det\left(I_{S_{k-1}}\left({\hat{\theta }}^{k-1}\right)+I_{i_{k}}\left({\hat{\theta }}^{k-1}\right)\right), \end{array}$$

where *S*_*k*−__1_ represents the set of *k*−1 administered items; *R*_*k*_ is the set of the remaining items in the item pool; *i*_*k*_ refers to the item administered as the *k*th item in the test; $I_{S_{k-1}}\left({\hat{\theta }}^{k-1}\right)$ and $I_{i_{k}}\left({\hat{\theta }}^{k-1}\right)$ refer to the two Fisher information matrices based on the specified item sets, and the ability estimate obtained from the first *k* −1 administered items,${\hat{\theta }}^{k-1}$. Bayesian A-optimality selects the *k*th item as

(6) $$\underset{i_{k}\in R_{k}}{\arg\min}\;\{\text{ trace }[\;{\left(I_{S_{k-1}}({\hat{\theta }}^{k-1})+I_{i_{k}}({\hat{\theta }}^{k-1})+\Sigma_{0}^{-1}\right]}^{-1}\},$$

where Σ_0_ refers to the prior covariance matrix of ability parameters.

Note that D-optimality, Bayesian D-optimality, A-optimality, and Bayesian A-optimality were commonly-used item selection criteria in MCAT (e.g., Diao and Reckase, 2009; Mulder and van der Linden, 2009; Chen and Wang, 2016; Chen et al., 2017). Bayesian A-optimality and D-optimality were inspected in the simulation. Bayesian A-optimality was used in the MCAT test due to its good performance in the ability recovery and item exposure (e.g., Diao and Reckase, 2009; Mulder and van der Linden, 2009; Ye and Sun, 2018). A-optimality tended to favor the items measuring relatively fewer latent traits in the test, which inevitably results in quite a few items measuring *K* traits are not administered (e.g., Diao and Reckase, 2009; Mulder and van der Linden, 2009; Ye and Sun, 2018), so A-optimality was not inspected in the simulation. In addition, due to the similar performance of D-optimality and Bayesian D-optimality in the pilot study by Ye and Sun (2018), the latter was not considered here. Other item selection methods in MCAT such as minimum angle (Reckase, 2009) and so on were not inspected for simplicity.

#### Test Lengths in MCAT

The fixed-length stopping rule was considered in the MCAT test for both the two studies. Each test was terminated when *Z*_0_ operational items were administered. Firstly, for both Study 1 and Study 2, the test length was designed as *Z*_0_ = 50. The number of replenished items administered in each test was designed as *Z*_1_ = 6 for Study 1 and *Z*_1_ = 7 for Study 2, respectively. Thus, the above tests were designed across eight conditions: two types of latent trait correlation (independent and correlated), two types of item discrimination (moderate and high) and two types of item selection criteria (D-optimality and Bayesian A-optimality).

Furthermore, this research also attempts to answer the question that how a short fixed-length test with highly discriminating items influences the performance of the LPRM, so Z_0_ = 25 operational items were designed in the tests for the two studies across four conditions: two types of latent trait correlation (independent and correlated), and two types of item selection criteria (D-optimality and Bayesian A-optimality). The number of administered replenished items was also *Z*_1_ = 6 for Study 1 and *Z*_1_ = 7 for Study 2, respectively.

#### Assignment of Replenished Items

In Study 1, all the replenished items in the item pool of MCAT were divided into 5 groups. Each group contained *Z*_1_ = 6 items, every two of which corresponded to one of the three different patterns. For each three-dimensional test in Study 2, all the replenished items in the item pool were also divided into 5 groups. Each group contained *Z*_1_ = 7 items, which corresponded to seven different patterns, respectively. For both the two studies, the examinees were equally divided into 5 groups to answer the 5 groups of replenished items, respectively. Thus, each replenished item was allocated to *n* = 400 examinees. Because all the patterns of replenished items were assumed unknown, examinees' responses to the replenished items were collected via MCAT and prepared for Step 2 of the LPRM.

#### Tuning Parameter Setting

As mentioned in the third section, the alternative λ values for Step 2 and Step 3 of the LPRM should be given sufficiently to make sure the alternative item-trait patterns yielded by the LASSO are diversely enough. Nevertheless, it is certainly not necessary for λ to take too many values, which probably lead to redundant alternative optimizations in LPRM. Thus, in order to give the λ values appropriately for the above two studies, several trials were made beforehand. The algorithm of cyclical coordinate descent was used. The details for setting λ values were designed as follows: An interval (0, *T*] was given and then was divided equally into *W* parts. The *W*-1 equidistant points and the right endpoint *T* of the interval were taken as λ_1_, …, λ_*W*_, respectively. For both the two simulation studies, the interval and its equidistant points were carefully tried for several times and taken as *T* = 120 and *W* = 80. Step 2 was implemented to get alternative patterns for all the replenished items under the λ values; the BIC was used to choose the optimal patterns from the alternatives, as introduced in Step 3.

### Evaluation Indices

#### Correct Specification Rate

To evaluate the pattern recognition accuracy of the LPRM, the correct specification rate (CSR) of the item-trait patterns of the overall replenished items was analyzed as

(7) $$\begin{array}{l} CSR=\frac{1}{J_{1}\times K}\overset{J_{1}}{\underset{j=1}{\sum }}\overset{K}{\underset{k=1}{\sum }}I\left(q_{jk}^{\text{*}}=q_{jk}\right). \end{array}$$

The vector ***Q***_*j*_ $={\left(q_{j1},\ldots ,q_{jK}\right)}^{\text{T}}$ represents the true pattern of the *j*th replenished item, while the vector ${\hat{Q}}_{j}^{*}={\left(q_{j1}^{*},\;\ldots ,\;q_{jK}^{*}\right)}^{T}$ represents the optimal pattern detected by the LPRM. Both *q*_*jk*_ and $q_{jk}^{*}$ takes 1 or 0, indicating whether the *k*th latent trait was measured by item *j* or not. The function *I* denotes a 0-1 valued indicator of whether the measurement relationship between the *j*th item and the *k*th trait is correctly specified by the LPRM or not.

#### Ability Estimation Accuracy

The absolute mean error (AME) and the root mean squared error (RMSE) for each of the 50 replications are calculated to evaluate the ability estimation accuracy of the examinees in the MCAT test. The two indices are defined in Equations (8, 9).

(8) $$\begin{array}{l} AME\left(\theta \right)=\frac{\overset{N}{\underset{i=1}{\sum }}\overset{K}{\underset{k=1}{\sum }}|{\hat{\theta }}_{ik}-\theta_{ik}|}{N\times K}, \end{array}$$

(9) $$\begin{array}{l} RMSE\left(\theta \right)=\sqrt{\frac{\overset{N}{\underset{i=1}{\sum }}\overset{K}{\underset{k=1}{\sum }}{\left({\hat{\theta }}_{ik}-\theta_{ik}\right)}^{2}}{N\times K}}. \end{array}$$

#### Item Parameter Estimation Accuracy

The estimation accuracy of the item parameters of replenished items is evaluated by the AME for each replication. Note that the item parameters were estimated based on the patterns identified by the LPRM with the known abilities: ability estimates from the MCAT and true abilities, individually. The AME for discrimination parameters and intercept parameters are defined in Equations (10,11).

(10) $$\begin{array}{l} AME\left(a\right)=\frac{\overset{J_{1}}{\underset{j=1}{\sum }}\overset{K}{\underset{k=1}{\sum }}|{â}_{jk}-a_{jk}|}{J_{1}\times K}, \end{array}$$

(11) $$\begin{array}{l} AME\left(b\right)=\frac{\overset{J_{1}}{\underset{j=1}{\sum }}|{\hat{b}}_{j}-b_{j}|}{J_{1}}. \end{array}$$

#### Item Exposure Indices

It seems that the LPRM is not directly affected by the item exposure. Nevertheless, it is necessary to screen whether severe overexposure of the operational items is potentially caused by the simulated conditions, especially those are propitious to provide more accurate estimates for Step 1 of the LPRM and benefit Step 2 of the LPRM. Since Step 1 of the LPRM depends on the MCAT to collect essential examinee information, an ideal situation can be expected as the more accurate estimates the LASSO gets, the better pattern recognition accuracy the LPRM shows. Therefore, after inspecting the pattern recognition accuracy and the ability recovery based on the above indices for the LPRM, the chi-square statistic (Chang and Ying, 1999) and test overlap ratio (TOR) (Chen et al., 2003) were calculated for assessing the item exposure of the operational items in the item pool in Studies 1 and 2. The chi-square statistic is defined as

(12) $$\begin{array}{l} \overset{J_{0}}{\underset{j=1}{\sum }}\frac{{\left[ER_{j}-Z_{0}/J_{0}\right]}^{2}}{Z_{0}/J_{0}}, \end{array}$$

where *ER*_*j*_ is the observed exposure rate for the *j*th item. The TOR is defined as:

(13) $$\begin{array}{l} \frac{N\times \overset{J_{0}}{\underset{j=1}{\sum }}{\left(ER_{j}\right)}^{2}}{\left(N-1\right)\times Z_{0}}-\frac{1}{N-1}. \end{array}$$

### Results of Studies 1 and 2

#### Pattern Recognition Accuracy of Replenished Items in Study 1

Table 1 lists the CSR values for the detected item-trait patterns of the replenished items in the two-dimensional item pool. Specifically, the CSR values across the first two columns indicated the performance of the LPRM with examinees' ability estimates in Step 2, while the CSR values across the last column indicated that of the LPRM with true abilities, which were regarded as benchmarks since the true abilities were believed to produce more precise patterns for the LPRM than the ability estimates.

**Table 1:** Correct specification rate of the item-trait patterns identified by the LPRM in Study 1.

**Condition**			**LPRM with ability estimates**		
**Item pool type**	**Test length**	**Latent trait correlation**	**Item selection criterion**		
			**D-optimality (%)**	**Bayesian A-optimality (%)**	**LPRM with true abilities (%)**
Items with moderate discrimination	50	Independent	85.20	83.10	90.37
	50	Correlated	92.10	92.07	94.23
Items with high discrimination	50	Independent	99.40	99.47	99.80
	50	Correlated	99.67	99.50	99.63
	25	Independent	99.30	99.33	99.80
	25	Correlated	99.60	99.40	99.63

As shown in Table 1, when examinees' ability estimates were used in Step 2 of the LPRM, the CSR was best under the condition of correlated abilities along with 50 highly discriminating items selected in the MCAT test. The CSR values under the three conditions with highly discriminating items were close to those under the above condition. For the four conditions with highly discriminating items, it was also of interest that the CSR values for the test with 25 operational items were even similar to that for the test with 50 operational items, which suggested the comparatively short test length did not remarkably decrease the pattern recognition accuracy of the LPRM. The CSR values under the two conditions of moderately discriminating items were lower than those under the other four conditions.

As for comparing the pattern recognition accuracy of the LPRM between the two types of trait correlation, Table 1 showed the CSR values for correlated-ability conditions were generally higher than those for independent-ability conditions, which is especially obvious for the item pool with moderately discriminating items. Table 1 also showed that D-optimality and Bayesian A-optimality performed similarly, and both the two criteria generally help to promote the LPRM had relatively high CSR values.

In addition, Table 1 showed the CSR values for the LPRM using ability estimates were close to their benchmarks except for two values at the first row. Note that for the fourth row of the table, the performance of the LPRM with true abilities, the benchmark, was slightly worse than that of the LPRM with ability estimates, which were obtained by the MCAT test with D-optimality. That phenomenon was probably due to the relatively small measurement error.

#### Ability Parameter Estimation Comparison in MCAT of Study 1

To validate the above results, the AME and RMSE values of the abilities from the fixed-length MCAT test were averaged across 50 replications and listed in Table 2. It showed that the AME and RMSE values for Bayesian A-optimality were slightly smaller than but close to those for D-optimality. For both the two criteria, the AME and RMSE values for the test with 50 highly discriminating items were generally smaller than those under the other four conditions.

**Table 2:** Recovery of the ability parameters in Study 1.

**Condition**			**Item selection criterion**			
**Item pool type**	**Test length**	**Latent trait correlation**	**D-optimality**		**Bayesian A-optimality**	
			**AME (θ)**	**RMSE (θ)**	**AME (θ)**	**RMSE (θ)**
Items with moderate discrimination	50	Independent	0.2546	0.3199	0.2503	0.3146
	50	Correlated	0.2517	0.3163	0.2492	0.3134
Items with high discrimination	50	Independent	0.2037	0.2572	0.2000	0.2527
	50	Correlated	0.2027	0.2562	0.2002	0.2529
	25	Independent	0.2786	0.3518	0.2749	0.3470
	25	Correlated	0.2752	0.3476	0.2731	0.3448

As shown in Table 2, it was of interest that the AME and RMSE values of the ability estimates for the test with 25 highly discriminating items were larger than those with 50 moderately discriminating items. That was distinct from the phenomenon shown by the CSR values in Table 1. It could be inferred that the pattern recognition accuracy of the LPRM was influenced more significantly by the item discrimination than the test length. Furthermore, two reasons probably help to explain the distinction: one was that high discrimination led to comparatively precise ability estimates, which improved the performance of the LPRM; the other was that large differences of the discrimination parameters between the measured and un-measured traits for a given replenished item were beneficial for the pattern recognition. Table 2 also showed that the ability recovery for the correlated conditions was generally slightly better than that for the independent conditions.

#### Item Parameter Estimation Accuracy of Replenished Items in Study 1

To further evaluate how the item-trait patterns identified by the LPRM affect the item parameter estimation accuracy, the item parameters of the replenished items were estimated based on the patterns identified by the LPRM in Study 1. The AME values of the estimated discrimination parameters and the intercept parameters were averaged across 50 replications and shown in Table 3. It indicated that the AME values for the estimated discrimination parameters were different, which can be explained by the distinct CSR values of the replenished items in Table 1. By computing the correlation between the CSR and AME in Tables 1, 3, it can be found that the CSR values of the identified item-trait patterns of the replenished items and the estimation accuracy of the discrimination parameters were highly negatively correlated, which suggested that the performance of the recovery of the discrimination parameters were significantly affected by the pattern recognition accuracy. Table 3 also showed that the AME values for the estimated intercept parameters were close to each other across all the conditions, which suggested that the estimation accuracy of intercept parameters was not as sensitive as the discrimination parameters to the pattern recognition accuracy.

**Table 3:** Recovery of the item parameters based on the item-trait patterns from the LPRM in Study 1.

**Condition**			**LPRM with ability estimates**					
**Item pool type**	**Test length**	**Latent trait correlation**	**Item selection criterion**					
			**D-optimality**		**Bayesian A-optimality**		**LPRM with true abilities**	
			**AME(*a*)**	**AME(*b*)**	**AME(*a*)**	**AME(*b*)**	**AME(*a*)**	**AME(*b*)**
Items with moderate discrimination	50	Independent	0.1927	0.1079	0.2110	0.1091	0.1498	0.1021
	50	Correlated	0.1387	0.1014	0.1410	0.1004	0.1197	0.0992
Items with high discrimination	50	Independent	0.0960	0.0954	0.0951	0.0953	0.0931	0.0946
	50	Correlated	0.0934	0.0975	0.0961	0.0979	0.0943	0.0992
	25	Independent	0.1004	0.0929	0.0998	0.0939	0.0931	0.0946
	25	Correlated	0.0975	0.0965	0.0983	0.0984	0.0943	0.0992

In addition, the AME values of the estimated discrimination parameters based on the item-trait patterns from the LPRM with true abilities were generally smaller than those with ability estimates. The values of the estimated intercept parameters for the LPRM with true abilities were slightly lower than those for the LPRM with ability estimates.

#### Item Exposure of the Operational Items in Study 1

As mentioned above, it is necessary to inspect whether the simulated conditions yield severe overexposure of the operational items in the item pool. Table 4 lists the corresponding chi-square and TOR values. It showed that D-optimality performed slightly better than Bayesian A-optimality in terms of the item exposure. Also, it showed that for both D-optimality and Bayesian A-optimality, the two conditions of selecting 50 highly discriminating items in the test could avoid the overexposure better than the other four conditions. Recalling that Table 1, 2 revealed the MCAT test with 50 highly discriminating items can provide relatively accurate ability estimates to the LPRM. Therefore, the conditions across enough length, high item discrimination, and the two item selection criteria used in an MCAT test were propitious to improve the performance of the LPRM and did not yield severe overexposure of the operational items, which potentially supported the feasibility of the LPRM in practice.

**Table 4:** Item exposure of the operational items in Study 1.

**Condition**			**Item selection criterion**			
**Item pool type**	**Test length**	**Latent trait correlation**	**D-optimality**		**Bayesian A-optimality**	
			**Chi-square**	**TOR**	**Chi-square**	**TOR**
Items with moderate discrimination	50	Independent	293.4326	0.3813	305.6727	0.3949
	50	Correlated	284.3236	0.3712	300.4173	0.3890
Items with high discrimination	50	Independent	229.2812	0.3100	238.6558	0.3204
	50	Correlated	222.5691	0.3025	235.2011	0.3165
	25	Independent	264.8699	0.3217	270.9162	0.3285
	25	Correlated	257.3573	0.3134	266.2580	0.3233

#### Pattern Recognition Accuracy of Replenished Items in Study 2

For the three-dimensional item pool, Table 5 lists the CSR for the LPRM with the fixed-length test under different conditions. When Step 2 of the LPRM used ability estimates via MCAT, it showed that the test selecting 50 highly discriminating items by either D-optimality or Bayesian A-optimality helped the LPRM produce the highest CSR values. The CSR values for the test with 25 highly discriminating items were close to those for the above two conditions, while those for the other two conditions with moderate item discrimination were significantly lower. The CSR values for the condition with correlated abilities and moderate item discrimination were higher than that with independent abilities. Also, the D-optimality and Bayesian A-Optimality performed similarly to each other with respect to influencing the pattern recognition accuracy of the LPRM. In addition, the CSR values for the four conditions with high item discrimination were quite close to their benchmarks, while those for the two conditions with moderate item discrimination were lower than their benchmarks.

**Table 5:** Correct specification rate of the item-trait patterns identified by the LPRM in Study 2.

**Condition**			**LPRM with ability estimates**		
**Item pool type**	**Test length**	**Latent trait correlation**	**Item selection criterion**		
			**D-optimality (%)**	**Bayesian A-optimality (%)**	**LPRM with true abilities (%)**
Items with moderate discrimination	50	Independent	85.22	83.75	90.42
	50	Correlated	92.90	92.55	99.14
Items with high discrimination	50	Independent	99.01	99.05	99.43
	50	Correlated	99.24	99.24	99.35
	25	Independent	98.78	98.82	99.43
	25	Correlated	98.74	98.61	99.35

#### Ability Parameter Estimation Accuracy in MCAT of Study 2

The AME and RMSE values of the abilities in Study 2 were listed in Table 6. It showed that Bayesian A-optimality performed slightly better than D-optimality in the ability recovery for the three-dimensional test. The abilities could be estimated more precisely for the conditions of correlated abilities than for those of independent abilities. For both the two item selection criteria, the AME and RMSE values for the two conditions of 50 highly discriminating items were better than those for the other four conditions. Although the ability recovery for the test with 25 highly discriminating items was worse than that for the test with moderate item discrimination, the CSR values shown in Table 5 indicated the pattern recognition accuracy for the former was better than that for the latter. It suggested that for the three-dimensional MCAT test, item discrimination influenced the pattern recognition accuracy for the LPRM more than the test length.

**Table 6:** Recovery of the ability parameters in Study 2.

**Condition**			**Item selection criterion**			
**Item pool type**	**Test length**	**Latent trait correlation**	**D-optimality**		**Bayesian A-optimality**	
			**AME (θ)**	**RMSE (θ)**	**AME (θ)**	**RMSE (θ)**
Items with moderate discrimination	50	Independent	0.2922	0.3675	0.2889	0.3634
	50	Correlated	0.2883	0.3632	0.2863	0.3609
Items with high discrimination	50	Independent	0.2376	0.3000	0.2343	0.2956
	50	Correlated	0.2363	0.2995	0.2345	0.2969
	25	Independent	0.3233	0.4077	0.3200	0.4037
	25	Correlated	0.3176	0.4018	0.3150	0.3990

#### Item Parameter Estimation Accuracy of Replenished Items in Study 2

For the three-dimensional item pool, the AME values of the estimated discrimination parameters, based on the patterns from the LPRM, and the intercept parameters of the replenished items were averaged across 50 replications and listed in Table 7. Similar to the results of Table 3, the AME values for the estimated intercept parameters in Table 7 were not obviously affected by the various conditions, which suggested that the estimation accuracy of intercept parameters was not much sensitive to the pattern recognition accuracy. Similar to the findings of Study 1, the estimation accuracy of the discrimination parameters was significantly negatively correlated with the pattern recognition accuracy of the replenished items.

**Table 7:** Recovery of the item parameters based on the item-trait patterns from the LPRM in Study 2.

**Condition**			**LPRM with ability estimates**					
**Item pool type**	**Test length**	**Latent trait correlation**	**Item selection criterion**					
			**D-optimality**		**Bayesian A-optimality**		**LPRM with true abilities**	
			**AME (*a*)**	**AME (*b*)**	**AME (*a*)**	**AME (*b*)**	**AME (*a*)**	**AME (*b*)**
Items with moderate discrimination	50	Independent	0.1829	0.1144	0.1957	0.1124	0.1393	0.1074
	50	Correlated	0.1304	0.1015	0.1330	0.1019	0.0660	0.0825
Items with high discrimination	50	Independent	0.0944	0.1024	0.0935	0.1011	0.0865	0.1017
	50	Correlated	0.0995	0.1077	0.0998	0.1080	0.0970	0.1069
	25	Independent	0.0988	0.1029	0.0993	0.1036	0.0865	0.1017
	25	Correlated	0.1086	0.1057	0.1077	0.1024	0.0970	0.1069

Besides, the AME values of the estimated discrimination parameters based on the item-trait patterns from the LPRM with true abilities were smaller than those with ability estimates. The AME values of the estimated intercept parameters for the LPRM with true abilities were close to those with ability estimates.

#### Item Exposure of the Operational Items in Study 2

The chi-square statistic and TOR values were listed in Table 8 to assess the item exposure of the operational items in Study 2. The results showed in this table were similar to those in Table 4. The two item exposure indices for the test with 50 highly discriminating items were lower than those for the other four conditions; D-optimality performed slightly better than Bayesian A-optimality in terms of the item exposure.

**Table 8:** Item exposure of the operational items in Study 2.

**Condition**			**Item exposure**			
**Item pool type**	**Test length**	**Latent trait correlation**	**Item selection criterion**			
			**D-optimality**		**Bayesian A-optimality**	
			**Chi-square**	**TOR**	**Chi-square**	**TOR**
Items with moderate discrimination	50	Independent	286.5815	0.3696	291.1572	0.3746
	50	Correlated	257.0860	0.3371	270.8551	0.3523
Items with high discrimination	50	Independent	219.7164	0.2960	228.6465	0.3059
	50	Correlated	197.7395	0.2719	214.0958	0.2899
	25	Independent	265.0295	0.3184	262.3904	0.3155
	25	Correlated	241.4214	0.2924	245.8582	0.2973

## Simulation for the LPRM With the Variable-Length MCAT

Note that for a fixed-length test, the estimation precisions of different examinees' abilities are often distinct. To achieve the same level of precision, the examinees may need varying test length, especially for those in the computerized adaptive testing (e.g., Choi et al., 2010; Yao, 2013; Wang et al., 2018). As pointed out by the reviewers of this paper, although the fixed-length stopping rule is easy to implement in large-scale administration, it often produces higher measurement errors at extreme trait levels due to premature termination. Also, it may reduce the efficiency of an MCAT due to potential unnecessary administration of items that contribute little information about an examinee's trait level (e.g., Choi et al., 2010). The variable-length rule (e.g., Boyd et al., 2010) intends to achieve approximately equal precision for all examinees by varying the number of items administered to each examinee, which is probably more efficient than the fixed-length rule.

Thus, Study 3 was conducted to explore the performance of the LPRM integrated with the variable-length MCAT. Except for the test length, Study 3 was designed to follow most of the settings and conditions in Studies 1 and 2 so that the performance of the LPRM could be conveniently compared between the fixed-length and variable-length MCAT scenarios.

### Data Generation, Test Design, and Evaluation Indices

In this study, the variable-length stopping rule in MCAT of the LPRM was designed as the standard error (SE) method (e.g., Weiss and Kingsbury, 1984; Boyd et al., 2010). For each examinee, the test was terminated when the SE values of ability estimates reached no more than 0.3. The maximum number of the operational items answered by each examinee was restricted at 100 to avoid the test was too long. The designs for the dimensionality of the items pools item discrimination, latent trait correlation, assignment of replenished items, and tuning parameter setting in Study 3 were as same as those in Studies 1 and 2. The item selection criterion for the MCAT was Bayesian A-optimality. The indices in Equations (7–11) for evaluating the pattern recognition accuracy of replenished items and the recovery of ability and item parameters were also inspected in Study 3. For simplicity, the D-optimality method and item exposure of the operational items were not considered in this study.

### Results of Study 3

#### Pattern Recognition Accuracy of Replenished Items in Study 3

Table 9 lists CSR values of the item-trait patterns of replenished items identified by LPRM in Study 3. By comparing the results under the same conditions among Tables 1, 5, 9 it can be found that for the two-dimensional item pool with moderately discriminating items, the CSR values for the LPRM with the variable-length test were slightly better than that with the fixed-length test. For the three-dimensional item pool with moderately discriminating items, the values for the variable-length rule were better than that for the fixed-length rule. For the two-dimensional and three-dimensional item pools with highly discriminating items, the values for the variable-length rule were close to those for the fixed-length rule.

**Table 9:** Correct specification rate of the item-trait patterns identified by the LPRM in Study 3.

**Condition**			**LPRM with ability estimates**
**Number of latent trait dimensions**	**Item pool type**	**Latent trait correlation**	**Bayesian A-optimality (%)**
Two	Items with moderate discrimination	Independent	84.27
		Correlated	92.57
	Items with high discrimination	Independent	99.43
		Correlated	99.47
Three	Items with moderate discrimination	Independent	87.01
		Correlated	93.28
	Items with high discrimination	Independent	98.97
		Correlated	99.26

#### Ability Parameter Estimation Accuracy in MCAT of Study 3

Table 10 lists the recovery of ability parameters estimated by the variable-length MCAT in Study 3. The comparison of the values among Tables 2, 6, 10 indicated that the variable-length rule in MCAT like the SE method controlled the ability estimation accuracy better than the fixed-length rule. The AME and RMSE values in Table 10 were consistently around 0.23 and 0.29, respectively, whereas the values for the fixed-length rule in Tables 2, 6 were affected significantly by item discrimination.

**Table 10:** Recovery of the ability parameters in Study 3.

**Condition**			**Item selection criterion**	
**Number of latent trait dimensions**	**Item pool type**	**Latent trait correlation**	**Bayesian A-optimality**	
			**AME (θ)**	**RMSE (θ)**
Two	Items with moderate discrimination	Independent	0.2372	0.2978
		Correlated	0.2356	0.2957
	Items with high discrimination	Independent	0.2371	0.2988
		Correlated	0.2355	0.2966
Three	Items with moderate discrimination	Independent	0.2367	0.2972
		Correlated	0.2349	0.2954
	Items with high discrimination	Independent	0.2344	0.2954
		Correlated	0.2311	0.2912

#### Item Parameter Estimation Accuracy of Replenished Items in Study 3

Table 11 lists the recovery results of item parameters of replenished items in Study 3, which were estimated based on the item-trait patterns from the LPRM with the variable-length MCAT. For the two-dimensional and three-dimensional item pools with moderately discriminating items, the AME values of the discrimination parameters for the variable-length rule were better than those for the fixed-length cases. For the two types of item pools with highly discriminating items, the AME values for the variable-length rule were close to those for the fixed-length rule. As for the recovery of intercept parameters, the AME values in Table 11 were slightly better than or close to those in Tables 3, 7.

**Table 11:** Recovery of the item parameters based on the item-trait patterns from the LPRM in Study 3.

**Condition**			**LPRM with ability estimates**	
**Number of latent trait dimensions**	**Item pool type**	**Latent trait correlation**	**Bayesian A-optimality**	
			**AME (*a*)**	**AME (*b*)**
Two	Items with moderate discrimination	Independent	0.2007	0.1074
		Correlated	0.1357	0.0999
	Items with high discrimination	Independent	0.0970	0.0946
		Correlated	0.0969	0.0989
Three	Items with moderate discrimination	Independent	0.1691	0.1092
		Correlated	0.1272	0.1003
	Items with high discrimination	Independent	0.0952	0.1028
		Correlated	0.0994	0.1046

#### Summary

In Study 3, the varying lengths of the MCAT tests under the condition of Bayesian A-optimality were also inspected. The comparison of the test lengths for examinees between the 50-length and variable-length rules can indicate which one of the two rules is more efficient, because the time for the ability estimation procedure via the 50-length MCAT takes up more than 70% of the computing time of the LPRM. Therefore, a too long test for the variable-length rule in MCAT means increasing both the time costs and risk of examinees' fatigue effect. The benchmark here for the test length in MCAT is set as 50. Under the variable-length rule, Study 3 showed that the test lengths for over 80% of the examinees under the two-dimensional item pool with moderately discriminating items were >50; those for over 90% of the examinees under the two-dimensional item pool with highly discriminating items were <50; those for all the examinees under the three-dimensional item pool with moderately discriminating items were >50; those for over 55% of the examinees under the three-dimensional item pool with highly discriminating items were <50.

Comprehensively considering the results of the pattern recognition accuracy, recovery of item parameters, and computing efficiency of the MCAT for ability estimation, it indicated that for the two-dimensional item pool with highly discriminating items, the variable-length rule in MCAT for the LPRM was a right choice. The reason for that was the comparatively short test length for most examinees and sufficient accuracy of ability estimates produced by the variable-length rule for the LPRM. For the three-dimensional item pool with highly discriminating items, the 50-length tests for the LPRM were better than the variable-length tests in terms of ensuring the pattern recognition accuracy and saving the computing time. For the two-dimensional and three-dimensional item pools with moderately discriminating items, the accuracy of identifying item-trait patterns and estimating item parameters for the LPRM with the variable-length tests were better than that with 50-length tests. However, the computing efficiency for variable-length tests was worse than that for 50-length tests. Thus, choosing an appropriate rule of the two for the LPRM should be judged and weighed by practitioners. Note that if the comparatively low computing efficiency of a long test produced by the variable-length rule in MCAT could be ignored in practice, the variable-length rule should be a good choice for the LPRM under the conditions that were inspected in Study 3.

## Conclusion and Discussion

This research proposes a data-driven method to search for the optimal item-trait patterns of the replenished items in the MCAT item pool. The idea of the proposed three-step method, the LPRM, is summarized as follows. The essential examinee information required by the LPRM is collected via an MCAT test, for which it is assumed that a compensatory MIRT model, such as the M2PLM, fits the items in the item pool. The LASSO, regarded as one of the most popular variable selection methods in multivariate regression analysis, is treated as another essential part of the LPRM. The BIC is applied to select the optimal patterns of the replenished items from the alternatives, obtained from the L_1_-regularized optimizations with different tuning parameters. Three studies under the conditions across various latent trait correlation, item discrimination, item selection criteria, and stopping rules were conducted in the simulation section to investigate the performance of the LPRM.

### Conclusion

Conclusion of identifying the item-trait patterns of replenished items in MCAT by the LPRM are drawn from both the theoretical analysis and the results of the three simulation studies: The CSR values for the LPRM were above 80% and almost above 90% for most of the inspected conditions, which indicates the LPRM can precisely detect the item-trait patterns of the replenished items in the two-dimensional and three-dimensional item pools. The results evidence that the LPRM can effectively implement the essential parts for the pattern recognition problem: it collects the response and ability information via an MCAT test, utilizes the LASSO for getting alternative item-trait patterns of the replenished items, and applies the BIC for determining the optimal patterns. Since the true ability parameters can never be known in practice, the results of the three studies suggest that the ability estimates from the MCAT test for the associated L_1_-regularized optimization could assist the LPRM effectively to identify the item-trait patterns accurately and efficiently.

The comparatively high discrimination for the items in the item pool takes a significantly positive effect on the pattern recognition accuracy of replenished items. The three studies suggest that the operational items with higher discrimination promote the LPRM getting better results in pattern recognition and item parameter estimation. The operational items with higher discrimination ensure the better ability recovery, which are propitious to improve the performance of the LPRM and do not yield severe overexposure of the operational items. Those potentially support the practical feasibility of the LPRM. Furthermore, as for the exploration of operating a relatively short fixed-length MCAT for the LPRM, the first two studies indicated that the number of the selected operational items with high discrimination could almost be reduced from 50 to 25, which does not dramatically decrease the pattern recognition accuracy of the LPRM.

Focusing on the comprehensive performance of the LPRM with the fixed-length and variable-length rules in pattern recognition accuracy, item parameter recovery and computing efficiency, the three studies suggest that for different types of dimensionality and item discrimination, none of the two types of rules has an absolute advantage to the other. Although the variable-length rule generally performs better in controlling ability estimation accuracy than the fixed-length rule, the LPRM is not much sensitive to the ability recovery for some of the inspected conditions. Specifically, for the two-dimensional item pool with highly discriminating items, the variable-length rule in MCAT is recommended for the LPRM because of its consistently good performance in pattern recognition accuracy, item parameter recovery and computing efficiency; by contrast, for the three-dimensional item pool with highly discriminating items, the 50-length rule is recommended. For both the two-dimensional and three-dimensional item pool with moderately discriminating items, there is a trade-off between the pattern recognition accuracy and computing efficiency for the two rules. Nevertheless, if the comparatively low computing efficiency of the long MCAT test produced by the variable-length rule can be ignored in practice, especially for the moderately discriminating item pool, the good performance of the LPRM under the rule in pattern recognition and item parameter recovery makes it a good choice for the LPRM.

For moderately discriminating item pool, the LPRM for the correlated abilities identifies better item-trait patterns and results in more accurate discrimination parameter estimates than those for the independent abilities. For the highly discriminating item pool, the LPRM for the correlated abilities performs similarly to that for the independent abilities. There is little difference in the recovery of the intercept parameters among all the inspected conditions.

### Discussion

Several advantages of the LPRM are summarized as follows: (1) The LPRM can accurately and efficiently detect the optimal item-trait patterns of replenished items even if the ability estimates are taken as a substitute for the true ability parameters for the LPRM. It means that the LPRM allows the inputs of the ability parameters of the M2PLM to have small measurement error, which makes it possible to utilize the examinees' essential information recorded in MCAT. That is sufficiently supported by the results of the three simulation studies, especially for the test with highly discriminating items. (2) The online feature of the MCAT, as used by Step 1 of the LPRM, can automatically put the item parameters of the replenished items on the same scale as the operational ones in the item pool, which avoids the factor rotation for the replenished items fitted by the M2PLM. Also, the MCAT designed for the LPRM does not require a very large sample size of examinees, e.g., the number of the examinees answering each replenished item is 400. The costs of time and labors for the pretest of the replenished items can be saved much due to the MCAT utilized by the LPRM. (3) The item-trait patterns detected by the LPRM can effectively improve the interpretability of the replenished items from the perspective of goodness-of-fit due to the advantage of the LASSO and the BIC for statistical variable selection: the sparsity in terms of item discrimination parameters yielded by the LASSO promotes the concision of the M2PLM; the convexity of the L_1_-regularized optimization greatly simplifies the corresponding computation; the application of the BIC provides the computational convenience for choosing the optimal patterns from the alternatives. The patterns of the replenished items detected by the LPRM, consequently, are potentially beneficial to the checking and modification of those items. (4) The design for the MCAT in the LPRM does not strictly require a fixed order between the operational and replenished items administered in the test. Thus, it allows the replenished items to be mixed into the operational ones in a relatively free or reasonable way such as that for content balancing. In addition, both the fixed-length and variable length stopping rule like the SE method for the MCAT test can be integrated with the LPRM.

Note that the LPRM can also be successfully implemented under higher ability dimensions such as the four and five dimensions for the conditions as same as those in the three studies. It is supported by the additional simulation, which is not shown here for simplicity. Compared with the three studies in this paper, it was found that the performance of the LPRM in pattern recognition accuracy and item parameter recovery of the replenished items for the four-dimensional and five-dimensional item pools are still good, although it can be slightly affected by the increased dimension numbers.

Future directions of the research include: (1) The research on the problem of item-trait pattern recognition may take into account how to extend the proposed method when content constraints are imposed upon item selection procedures (e.g., Veldkamp and van der Linden, 2002; Yao, 2014) or for the MCAT based on other variable-length stopping rules (e.g., Yao, 2013; Wang et al., 2018). (2) It is possible for the future study to consider other shrinkage methods for selecting appropriate patterns of replenished items such as elastic net (Zou and Hastie, 2005), adaptive LASSO (Zou, 2006), or smoothly clipped absolute deviation (SCAD; Fan and Li, 2001) with the proposed pattern recognition procedure. Those popular shrinkage methods are developed to overcome the disadvantages of the LASSO for variable selection in linear regression models and generalized linear models. Note that the LASSO may produce inconsistent coefficient estimates under certain scenarios, and have some shortcomings for very high correlation of predictors or for the condition of large number of predictors and small number of observations (e.g., Fan and Li, 2001; Zou and Hastie, 2005; Zou, 2006). In psychometrics, Yoo (2018) uses the elastic net with logistic regression to show how to select variables from a large number of predictors in the data analysis of educational large-scale tests. Therefore, although the proposed LASSO-based method shows success under the simulation situations of this paper (i.e., comparatively small ability dimension, and not strong correlation between abilities), future study can explore how the popular shrinkage methods perform with the proposed pattern recognition procedure under other scenarios in MCAT such as large ability dimensions, very high correlation between abilities, or strong multicollinearity in terms of abilities. (3) The proposed method can also be generalized for the items fitted by other MIRT models, such as the bi-factor models (Gibbons and Hedeker, 1992; Gibbons et al., 2007), the two-tier full-information item factor model (Cai, 2010), or the polytomous compensatory MIRT models. (4) A limitation of the proposed method is that its idea for pattern recognition constructs on the compensatory MIRT models, so how to extend that to suit for the non-compensatory MIRT models can be considered in the future.

## Data Availability

The datasets generated for this study are available on request to the corresponding author.

## Author Contributions

JS was in charge of the original idea of the study, the literature review, the methodology and computing codes, the simulation design, and the drafting and revision of the manuscript. ZY contributed to the literature review, the computing codes, the simulation design, program running, and the revision of the manuscript.

### Conflict of Interest Statement

The authors declare that the research was conducted in the absence of any commercial or financial relationships that could be construed as a potential conflict of interest.
